# Clearing the Fog of Anticancer Patents from 1993–2013: Through an In-Depth Technology Landscape & Target Analysis from Pioneer Research Institutes and Universities Worldwide

**DOI:** 10.1371/journal.pone.0103847

**Published:** 2014-08-01

**Authors:** Ajay Dara, Abhay T. Sangamwar

**Affiliations:** Department of Pharmacoinformatics, National Institute of Pharmaceutical Education and Research (NIPER), S.A.S. Nagar, Punjab, India; Katholieke Universiteit Leuven, Belgium

## Abstract

**Background:**

In a search for an effective anticancer therapy the R&D units from leading universities and institutes reveal numerous technologies in the form of patent documents. The article addressed comparative anticancer patent landscape and technology assessment of Council of Scientific and Industrial Research (CSIR): India’s largest R&D organisation with top twenty international public funded universities and institutes from eight different countries.

**Methodology/Principal Findings:**

The methodology include quantitative and qualitative assessment based on the bibliometric parameters and manual technology categorisation to understand the changing patent trends and recent novel technologies. The research finding analysed 25,254 patent documents from the year 1993 to 2013 and reported the insights of latest anticancer technologies and targets through categorisation studies at the level of drug discovery, development and treatment & diagnosis. The article has reported the technology correlation matrix of twelve secondary class technologies with 34 tertiary sub-class research area to identify the leading technologies and scope of future research through whitespaces analysis. In addition, the results have also addressed the target analysis, leading inventor, assignee, collaboration network, geographical distribution, patent trend analysis, citation maps and technology assessment with respect to international patent classification systems such as CPC, IPC and CPI codes.

**Conclusions/Significance:**

The result suggested peptide technology as the dominating research area next to gene therapy, vaccine and medical preparation containing organic compounds. The Indian CSIR has ranked itself at seventh position among the top 20 universities. Globally, the anticancer research was focused in the area of genetics and immunology, whereas Indian CSIR reported more patents related to plant extract and organic preparation. The article provided a glimpse of two decade anticancer scenario with respect to top public funded universities worldwide.

## Introduction

The burden of cancer among other diseases has become a menace to human beings globally. Cancer is reported as the second cause of common deaths after cardiovascular disease and became one of the leading threat worldwide [1]–[4]. Cancer is a chronic disease with the increasing demographic characteristics varying widely, reporting more than 28 type of cancer in 184 countries [5]–[8]. According to GLOBOCAN 2012, it is estimated that by 2025 the cancer mortality rate would progressively increase with more than 19.3 million new cancer cases registered every year [9]. The International Agency for Research on Cancer (IARC) and the specialized cancer agency of the World Health Organization (WHO) has released the latest 2012 year data revealing 8.2 million death out of 14.1 million new cases reported. Moreover, the under developed countries have been affected more reporting half of the cancers cases with the deaths rate of 64.9% in the year 2012, and this number may increase in near future [10]–[12]. The rise in the incidence of death rate has exacerbated the need for exploring the latest cancer technologies and various targets, so as to get a comprehensive overview of the global cancer scenario [13]–[15].

On the other hand, intense research is being carried out by the universities, institutes, public funded organisation and many other multinational companies in order to unveil the secret mysteries of cancer, but the remedy still remains uncertain [16], [17]. In this process, numerous patent documents get published and granted every day, unleashing the latest anticancer technologies and targets [18], [19]. We selected the top twenty international public funded universities and institutes from eight different countries and compared the trends with Council of Scientific and Industrial Research (CSIR): one of the India’s largest R&D organisation with more than 37 research institute, 4 units and 39 outreach centres [20]–[23]. The aim was to gather the patent information on cancer and analyse them to understand various targets and latest anticancer technologies, so as to identify the ranking of CSIR among the top international universities. Furthermore, we reported the leading institutes, scientist and their collaboration network along with their changing patent patterns and trends [24]–[26]. This landmark study should provide a unique glimpse of current cancer scenario with respect to public funded universities & research institutes and help identify the technology whitespace for future research.

## Methodology

The main objective of the research is to provide a comprehensive overview of the latest anticancer technologies through a “Patent landscape analysis” [27]–[29]. The text mining method was used for screening the relevant anticancer patents from Thomson Innovation database using keyword based search methodology [30]. At first, the various synonyms of cancer were identified and listed out by thorough literature survey and then the top twenty universities screened according to their subject pharmacy and pharmacology for the year 2013 (Table 1). The selected universities were considered from the website http://www.topuniversities.com/, which evaluate through the “star” rating, ranging from 0 to 5 stars using more than 50 different indicators together contributing the overall assessment [21], [31]. Then a query string was generated using all possible cancer synonyms including the selected universities as follows.

**Table 1:** List of top pharmaceutical universities selected for landscape studies.

Sl No.	List of University (Country)
1.	Harvard University (US)
2.	University Cambridge (UK)
3.	National University Singapore (SG)
4.	University Oxford (UK)
5.	Karolinska Institute (SE)
6.	Monash University (AU)
7.	Imperial College London (UK)
8.	University Tokyo (JP)
9.	University Melbourne (AU)
10.	University Michigan (US)
11.	University Toronto (CA)
12.	University Pennsylvania (US)
13.	University Queensland (AU)
14.	University Wisconsin Madison (US)
15.	Boston University (US)
16.	Kings College London (UK)
17.	University California (US)
18.	University Manchester (UK)
19.	Johns Hopkins University (US)
20.	University Washington (US)
21.	CSIR (IN)

Source: http://www.topuniversities.com/university-rankings/university-subject-rankings/2013/pharmacy.

CTB = ((Cancer Or Anti*1cancer Or Chemotherap* Or Oncol* Or Carcinog* Or Neoplas* Or Tumor Or Metastat* Or Malignan*) AND (Treatment OR treating OR diagnos* OR Prevent*)) AND PA = (((Council near5 Scientific near5 Industrial near5 Research) OR (CSIR) and (India)) OR (Harvard near5 University) OR (University near5 Cambridge) OR (National near5 University near5 Singapore) OR (University near5 Oxford) OR (Karolinska near5 Institute) OR (Monash near5 University) OR (Imperial near5 College near5 London) OR (The near5 University near5 Tokyo) OR (The near5 University near5 Melbourne) OR (University near5 Michigan) OR (University near5 Toronto) OR (University near5 Pennsylvania) OR (University near5 Queensland) OR (University near5 Wisconsin near5 Madison) OR (Boston near5 University) OR (Kings near5 College near5 London) OR (University near5 California) OR (University near5 Manchester) OR (Johns near5 Hopkins near5 University) OR (University near5 Washington))); Thomson Innovation Patent Export, 2013-12-20 00:27∶14 -0600.

The search was performed on 20^th^ Dec 2013 in the Title/Abstract/Claims (CTB) of the patent document using only the cancer string alone to retrieve 9,37,814 patents. Then to narrow down the results, filters such as selected 21 assignee and application year of 1993 was used to bring the patent count to 25,254 documents. A few more studies such as the keyword based technology analysis and clustering was performed using the PatBase software of Mine*Soft* and RWS group [32].

## Results and Discussion

The patent portfolio of 25,254 documents were subjected to special condensing filter of **In**ternational **Pa**tent **Do**cumentation **C**entre (INPADOC) to retrieve 1584 unique families, which include 1068 applications and 516 granted patents contributing to 67% and 33%, respectively. Then patent landscape studies were performed on these 1584 patent documents to understand the trends, leading assignee and inventor, collaboration analysis, core technologies and their correlation along with the various target analysis.

### Assignee analysis

**Figure 1:**
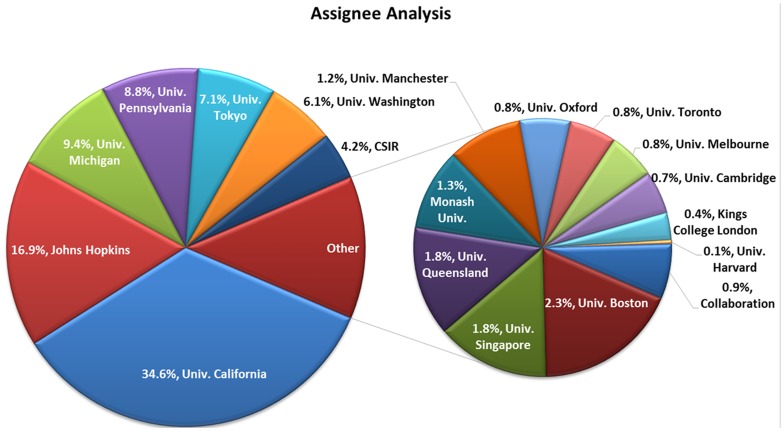
Assignee Analysis pie chart illustration.

The Figure 1 shows a pie chart representation of various assignees along with their percentile contribution. As per the given details, the University of California is the leading assignee reporting 548 patents contributing 34.6%, followed by Johns Hopkins, University of Michigan, University of Pennsylvania and University of Tokyo contributing each with 267 (16.9%), 149 (9.4%), 140 (8.8%) and 112 (7.1%), respectively. These six public funded research institutes and universities are reported as the major anticancer patent shareholders *i.e*. a total 1216 patent documents contributing 76.8%, respectively. The University of Washington is ranked at sixth with 97 anticancer patent contributing 6.1%. Whereas, The Indian CSIR has occupied the seventh position among the top 20 international universities by reporting 67 anticancer patents which contribute to 4.2% of overall 1584 patents. The University of Boston, University of Singapore and University of Queensland has showed 37, 29 and 28 patents, respectively contributing approximately 2% each. However, University of Monash and University of Manchester has reported 21 and 19 patents showing an approximate 1% contribution each. The remaining universities such as University of Oxford, University of Toronto, University of Melbourne, University of Cambridge, Kings College London and Harvard University includes the collaboration patents as well, reporting less than 1% patent contribution each. The geographical assignee analysis indicate that altogether there are 21 assignees selected from eight different countries including United States of America, United Kingdom, Canada, Singapore, Australia, Japan, Sweden and India. The country wise ranking of the universities illustrate that United States of America stands first covering 8 top universities followed by United Kingdom, Australia reporting 5 and 3 top institutes, respectively whereas other five countries contributed one top university each.

### Collaboration network analysis

The collaboration network analysis help understand internal and external joint ventures of one university with other institute or company etc. This study will narrow down the selection of potential assignees which are working in specific field of technology and likely to identify licensee. The collaboration network analysis have been studied at two different levels. One the internal collaboration network which includes collaboration within the selected 21 universities and other external collaboration indicating assignees other than selected, acting as joint patentees.

#### Internal collaboration network

**Figure 2:**
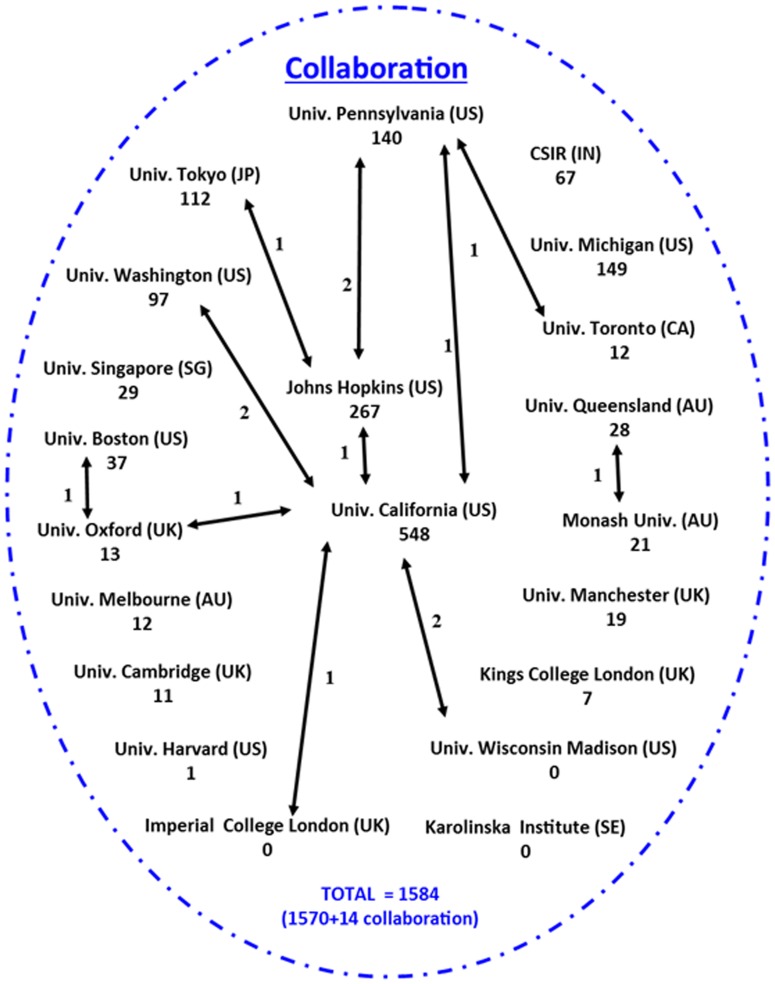
Internal assignee collaboration network analysis.

The Figure 2 indicates the internal collaboration network analysis from selected 21 universities, reporting the University of California as leading assignee with 8 collaboration patents contributing 2 each with University of Washington and Wisconsin Madison and other four are reported each with University of Pennsylvania, Imperial College London, Johns Hopkins and Oxford respectively. The second highest collaboration was shown by the University of Pennsylvania reporting 2 joint patents with Johns Hopkins and one each with University of California, Tokyo and Toronto. The University of Johns Hopkins stand at third position with altogether 4 joint patents, whereas the University of Boston and Oxford share one joint patents and University of Queensland and Monash share another joint patent. Altogether, out of the selected 21 public funded universities only 12 universities and institutes have shown internal collaboration within themselves to contribute a patent count of 14 *i.e.* 0.8% of total 1584 patents. A point to be noted that, University of Wisconsin Madison and Imperial College London share only the joint patent privilege, whereas Karolinska Institute neither has reported a joint patent nor contributed for an individual patent.

#### External collaboration network

Table 2 illustrate the external collaboration data with respect to 21 selected assignees, there are total 359 patent document reported with one or more external joint assignees along with the selected universities. The University of California is leading with 91 out of 548 patent documents showing one or more number of external assignees as joint partners. The University of Tokyo hold the second position with 70 external joint collaboration followed by Johns Hopkins, University of Washington, Pennsylvania, Michigan and Oxford reporting 62, 27, 22, 20 and 11 joint patents, respectively. The University of Singapore, Queensland and Monash reported 8 joint patents followed by University of Boston with 7 documents. The Indian CSIR stand at 12^th^ position showing five joint patent with Chitaranjan National Cancer Inst., Department of Biotechnology, Panjab University, Kakatiya University and Tufts University respectively. The University of Toronto showed 5 patents and University of Melbourne and Cambridge reported 4 each, followed by University of Manchester with 2 and finally Harvard University reporting the least of one patent as joint venture. Whereas, the other 4 internal joint patent documents were reported in collaboration by Astrazeneca, Ohio State Res. Foundation, Children’s Hospital of Philadelphia, University of Utah and Ludwig Inst. for Cancer Res. Ltd., respectively.

**Table 2:** External joint collaboration analysis.

University	External Joint Collaborations
Univ. California	91
Univ. Tokyo	70
Johns Hopkins	62
Univ. Washington	27
Univ. Pennsylvania	22
Univ. Michigan	20
Univ. Oxford	11
Univ. Singapore	8
Univ. Queensland	8
Monash Univ.	8
Univ. Boston	7
CSIR	5
Univ. Toronto	5
Univ. Melbourne	4
Univ. Cambridge	4
Univ. Manchester	2
Univ. Pennsylvania | Johns Hopkins	1
Monash Univ. | Univ. Queensland	1
Univ. California | Univ. Wisconsin Madison	1
Johns Hopkins Univ. | Univ. Tokyo	1
Univ. Harvard	1
**TOTAL**	**359**

Table 3 depicts the data of top 20 external joint assignees along with their patent count. The Oncotherapy Science Inc. is the leading joint assignee reporting 43 patents in collaboration mostly with University of Tokyo. The University of California and Johns Hopkins showed most collaboration with the U.S. Department of Health & Human Services accounting 13 patent documents. The Sanford-Burnham Inst. for Medical Research and Forerunner Pharma Res. Co. Ltd. reported 8 collaboration patents each and thereafter Leland Stanford Junior University, Perseus Proteomics Inc. and Massachusetts Inst. of Technology reported 5 joint patents each, respectively. The City of Hope, Cytochroma Inc., University of Arizona, Human Genome Sciences Inc. and University of Maryland reported 4 collaboration each followed by Genzyme Corporation, Nereus Pharmaceuticals Inc., Dana Farber Cancer Inst. Inc., Agency for Science & Res. and Brigham & Women’s Hospital Inc. covering each 3 patent documents. These external assignee not only covers the already mentioned eight geographical area, but also include the countries such as Denmark, Israel, France, Italy, Korea and Taiwan etc. There are more than 230 patent documents which reported only one or two joint external assignees accounting to a total of 359 external collaboration.

**Table 3:** List of top external joint assignee’s other than selected Universities.

Names of external joint assignee’s	Patent Count
Oncotherapy Science Inc.	43
Govt. of USA as Represented by Secretary of Dept. of Health & Human Services	13
Sanford-Burnham Inst. for Medical Res.	8
Forerunner Pharma Res. Co. Ltd.	8
Leland Stanford Junior Univ.	5
Perseus Proteomics Inc.	5
Massachusetts Inst. of Technology	5
City of Hope	4
Cytochroma Inc.	4
Univ. of Arizona	4
Univ. of Maryland	4
Human Genome Sciences Inc.	4
Genzyme Corporation	3
Nereus Pharmaceuticals Inc.	3
Dana Farber Cancer Inst. Inc.	3
Agency for Science & Res.	3
Brigham & Women's Hospital Inc.	3
Ludwig Inst. for Cancer Res.	3
Singapore Health Services Pte Ltd	2
Oncotherapy Science Inc. | National Univ. Corporation Gunma Univ.	2
Others	230
**TOTAL**	**359**

### Inventor analysis

The inventor analysis was performed on the 1584 patent documents to identify the leading scientist. The studies were performed at two different levels, one including all the inventors and other considering only the first inventor of the each patent document. Table 4 displays the information of top inventors and Nakamura, Yusuke from University of Tokyo/Oncotherapy Science Inc. is the leading inventor reported with more than 50 patents followed by the Nakatsuru, Shuichi from the same institute. Vogelstein, Bert and Kinzler, Kenneth, W. are the next active inventors from University of Johns Hopkins reporting 28 and 25 patents respectively. The Kamal, Ahmed a scientist from Indian CSIR has made it to occupy the fifth position at international level reporting 20 patents. The sixth position is taken by Wang, Shaomeng from University of Michigan with 18 patents followed by Carson, Dennis, A. and Gray, Joe, W. from University of California reporting 17 and 15 patents, respectively. The scientist Daigo, Yataro; Aburatani, Hiroyuki and Furukawa, Yoichi from University of Tokyo has contributed almost 15 patents each, following Greene, Mark, I. from University of Pennsylvania with 13 patents. Saxena, Ajit, Kumar another Indian scientist from CSIR has made it to the top 20 inventors list contributing 12 patent documents equalling the Kataoka, Kazunori and Katagiri, Toyomasa from University of Tokyo. Finally, the remaining five scientist Lenz, Heinz-Josef; Weiner, David, B; Jablons, David, M; Sidransky, David and Posner, Gary, H. are from Universities like Pennsylvania; Johns Hopkins and California.

**Table 4:** List of top Inventor analysis shown.

Rank	Inventor Name	University	Patent Count
1	Nakamura, Yusuke	Univ Tokyo	50
2	Nakatsuru, Shuichi	Univ Tokyo	29
3	Vogelstein, Bert	Johns Hopkins	28
4	Kinzler, Kenneth, W.	Johns Hopkins	25
5	Kamal, Ahmed	CSIR	20
6	Wang, Shaomeng	Univ Michigan	18
7	Carson, Dennis, A.	Univ California	17
8	Gray, Joe, W.	Univ California	15
9	Daigo, Yataro	Univ Tokyo	15
10	Aburatani, Hiroyuki	Univ Tokyo	15
11	Furukawa, Yoichi	Univ Tokyo	14
12	Greene, Mark, I.	Univ Pennsylvania	13
13	Saxena, Ajit, Kumar	CSIR	12
14	Kataoka, Kazunori	Univ Tokyo	12
15	Katagiri, Toyomasa	Univ Tokyo	12
16	Lenz, Heinz-Josef	Univ California	11
17	Weiner, David, B.	Univ Pennsylvania	10
18	Jablons, David, M.	Univ California	10
19	Sidransky, David	Johns Hopkins	9
20	Posner, Gary, H.	Johns Hopkins	9

**Figure 3:**
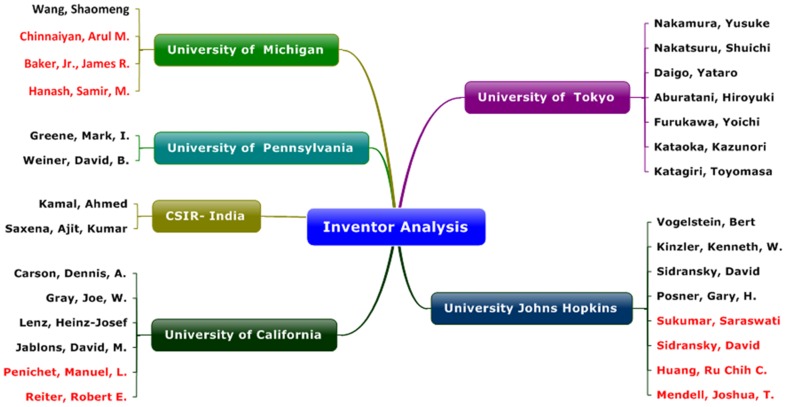
Inventor analysis Mindmap.

The first inventor analysis was also performed and few more leading scientist were identified and reported in the Table 5. Out of the top 20 first inventors, 11 scientists were already identified in the earlier inventor analysis and nine new names were added which are highlighted in ***bold italics*** font (Table 5). Sukumar, Saraswati from Johns Hopkins and Chinnaiyan, Arul M and Baker, Jr., James R from University of Michigan has reported 8 patents each as first inventors. Whereas, Sidransky, David and Hanash, Samir, M. accounted 7 patents each following Huang, Ru Chih C. from University of Johns Hopkins with 6 patents respectively. Lastly, Mendell, Joshua, T. from Johns Hopkins and Penichet, Manuel, L. and Reiter, Robert E. from University of California has reported 5 patents each as first inventors. Altogether, only three institutes i.e. University of Johns Hopkins, Michigan and California has reported the four, three and two new scientists respectively. Furthermore, a correlation study with respect to inventor vs. institute was performed through Mindmap (Figure 3) and identify University of Johns Hopkins and Tokyo as the leading institute which consider the inventors name for both assignee as well as inventor reporting almost 8 scientist out of top 29 inventors identified. Whereas, the University of California has reported 6 top inventors followed by University of Michigan with 4 scientist contributing to their labs respectively. Lastly, CSIR-India and University of Pennsylvania has two of the leading scientist in their labs. Altogether, the inventor analysis Mindmap through Figure 3 has reported and ranked the top 29 inventors screened out of 21 pioneer public funded research institutes.

**Table 5:** List of first Inventor analysis shown.

Rank	First Inventor	University	Patent Count
1	Nakamura, Yusuke	Univ Tokyo	45
2	Kamal, Ahmed	CSIR	20
3	Wang, Shaomeng	Univ Michigan	17
4	Carson, Dennis A.	Univ California	13
5	Aburatani, Hiroyuki	Univ Tokyo	12
6	Weiner, David, B.	Univ Pennsylvania	10
7	Vogelstein, Bert	Johns Hopkins	10
8	Greene, Mark, I.	Univ Pennsylvania	10
9	Lenz, Heinz-Josef	Univ California	9
10	***Sukumar, Saraswati***	***Johns Hopkins***	***8***
11	Posner, Gary H.	Johns Hopkins	8
12	Kataoka, Kazunori	Univ Tokyo	8
13	***Chinnaiyan, Arul M.***	***Univ Michigan***	***8***
14	***Baker, Jr., James R.***	***Univ Michigan***	***8***
15	***Sidransky, David***	***Johns Hopkins***	***7***
16	***Hanash, Samir, M.***	***Univ Michigan***	***7***
17	***Huang, Ru Chih C.***	***Johns Hopkins***	***6***
18	***Penichet, Manuel, L.***	***Univ California***	***5***
19	***Mendell, Joshua, T.***	***Johns Hopkins***	***5***
20	***Reiter, Robert E.***	***Univ California***	***5***

### Patent trend analysis

**Figure 4:**
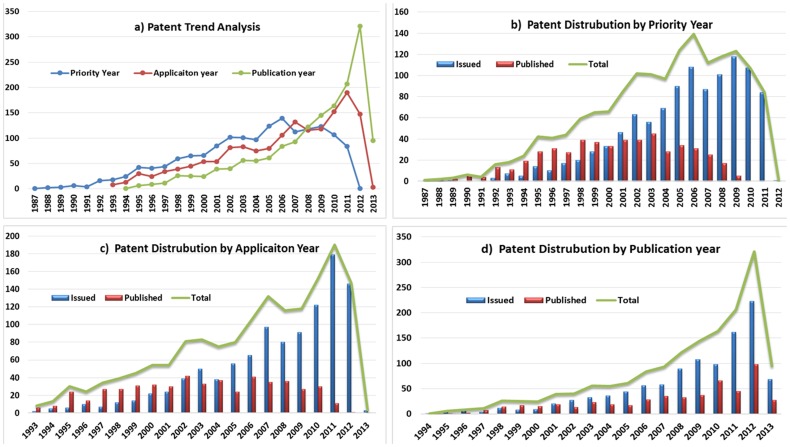
Patent trend distribution analysis based on issued and published documents.

To identify the public funded anticancer patent filing and granting pattern the patent trend analysis was performed at three different level *i.e.* the early priority, application and publication years. All the graphs are plotted taking year on *X*-axis and number of patents on *Y*-axis, whereas the combo graph of line and bar charts were selected to represent the trends. The Figure 4a depicts the comparative analysis of all the three trends in the form of a line graph representation. As per the graph the patent applications starting from the year 1993 to 2013 have claimed the earliest priority year of 1987–2012. The number of patents with respect to priority year have dominated followed by the application and publication year showing a overall progressive increase from the year 1993–2007. The maximum number of patent documents for the priority, application and publication year were reported to be 124, 190 and 321 for the years 2005, 2011 and 2012, respectively. The overall patent trend showed a normal progressive growth except some fluctuation in year 2008 which shifted the trend. The Figure 4b illustrates year wise published (purplish red) and issued (blue) patent distribution with respect to priority year. The combo graphs depict that from year 1987 to 1999 the number of published patents were more than issued documents and year 2000 reported the equal number of 33 patent documents for both categories. However, the trends seem to change from year 2001 to 2011 by showing a sharp increase in the number of issued documents to reach its maximum of 118 in 2009. Overall, the priority year trend has reported a normal distribution curve with an increasing trend from year 1987 to 2006 and then after a downfall reported till 2011.

Now considering the patent distribution with respect to application year (Figure 4c), the same trend seems to be followed reporting more published document from 1993 to 2002 and thereafter a vice-versa trend till 2012 to report its peak document of 190 issued in the year 2011. Whereas, the overall total distribution has showed and exponential growth rate from 1993 to 2012 reporting slight fluctuations. However, a changing trend can be observed with respect to patent distribution by publication year (Figure 4d), wherein the number of issued patent document have showed domination throughout the years 1994 to 2013. The year 1994 reported the least count of one and 2012 accounted the maximum count of 223 patent documents respectively. The overall sharp increasing trend was observed till 2012 with no reporting of downfall, so it’s expected to follow the same trend in near future. These four graphs with respect to priority, application and publication year conclude that the period 2005–2010 is the booming period for the anticancer patent filed and granted by the pioneer public funded research organisation. It also makes it clear that the maximum total patent count per year was reported by publication year followed by application and priority year. However, a vice-versa pattern is observed with respect to trend analysis from year 1987 to 2007 and the year 2008 has reported a shift in the ongoing trend by showing more number of published patents crossing the application and priority year documents.

### Geographical area analysis

**Figure 5:**
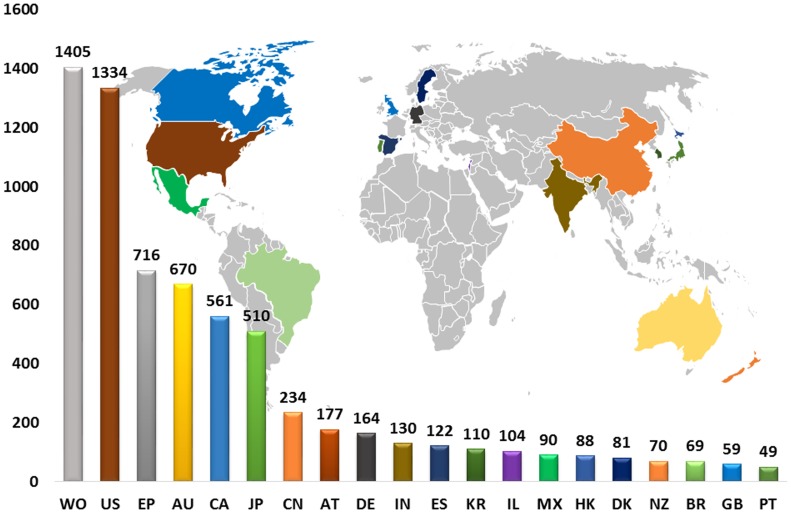
Patent Geographical Analysis.

Figure 5, illustrate the geographical areas covered by these 1584 patents represented in the form of bar graph and appropriately coloured in the world map. In geographical analysis each single patent document can be counted more than ones based on the number of countries it is filed and granted. So, the analysis results are based on the single application filed at different countries or at various national levels, in other words these applications are called as “also published as” patent documents. According to the given data, the maximum number of patent application are reported by the Patent Cooperation Treaty (PCT) applications covering 20.2% followed by United State of America and European Patent Office (EPO) application showing 19.2% and 10.3%, respectively. A total of 59 countries are involved in the geographical coverage, the top ten countries reported are as follows United States of America, Australia, Canada, Japan, China, Austria, Germany, India, Spain and Republic of Korea contributing 19.2%, 9.6%, 8.1%, 7.3%, 3.4%, 2.5%, 2.4%, 1.9%, 1.8% and 1.6%, respectively. Out of the top 10 countries reported most of them are from high human developed countries except India and China which are from developing nation. Altogether these ten countries along with PCT and EPO application have contributed more than 68% of total application. The remaining 32% are covered by other countries which includes Italy, Mexico, United Kingdom, Denmark, Norway, New Zealand, Hong Kong, Russia, Brazil and Taiwan etc. If we consider the continent wise patent distribution of these 18 reported countries excluding PCT and EPO application, 16.13% of Asia has been covered including 5 countries followed by 12.5% of Europe with 6 countries and Israel covered 4.76% of Middle East. However, USA, Canada and Mexico covered 75% of maximum area in North America, whereas, Australia and New Zealand covered 10% of Pacific countries and finally Brazil contributed 7.69% from South America.

### Patent citation analysis

The Patent citation studies was performed to identify the core technologies which are active in term of reference citations. We have selected three types of citation count for the study, the cited patents or backward citation, citing patents or forward citation and non-patent cited referees to identified the leading patent and explore their technology. The number of citation may vary based on the published document in different countries with same invention and title and also the citation count get updated time to time, hence the date of access is important.

#### Cited patent analysis

**Figure 6:**
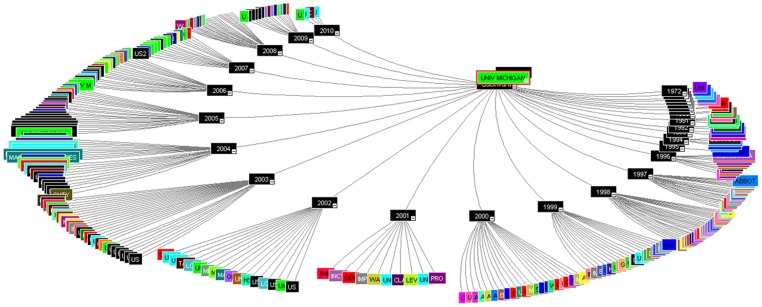
Cited patent analysis using Thomson Innovation.

The US patent (US8148147B2) of University of Michigan has reported the maximum backward citation of 302, the patent discuss about the “compositions and methods for treating and diagnosing pancreatic cancer” [33]. The patent revealed a new isolated population of cancer stem cells useful for studying, diagnosing, and treating solid tumours e.g. prostate cancer stem cells that are tumorigenic and positive for CD44, CD24, and epithelial-specific antigen (ESA). The patent was categorised under cell therapy technology and the document is also published as EP2106439A2, US20080261244, US20120135416, WO2008092002A2, US8148147 and WO2008092002A3. The patent document has been published in year 2013 claiming the earliest priority year 2007 and has cited the patents way back from 1972 of University of Leland Stanford Junior till 2010 of University of Michigan. The isolated pancreatic cancer stem cells and method are useful for treating a patient with pancreatic cancer in addition also useful for studying, diagnosing, and treating solid tumours (Figure 6).

#### Citing patent analysis

**Figure 7:**
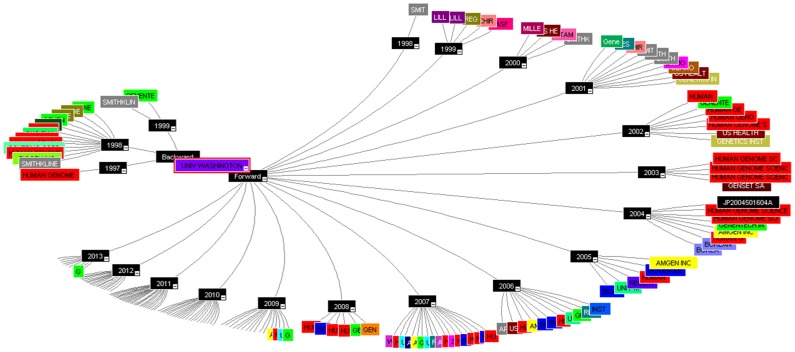
Citing patent analysis using Thomson Innovation.

The patent with maximum citing count is considered as the core technology, as many inventor/assignees are working in the same field of technology and the work is assumed to be actively performed based on number of forward citation. The PCT application WO1999011791A2 with a count of 168 citation has been reported as the top citing patents assigned to University of Washington with only one inventor Chaudhary Preet M. The patent was published in year 1999 claiming the priority year 1997 and talks about the invention related to the new tumour necrosis factor (TNF) family receptor polypeptides and ligands useful for diagnosis and treatment of prostate cancer and developmental or gestational abnormalities [34]. The novelty of the invention claims the isolated TNF polypeptides: apolipoprotein APO4, APO6, APO8 and APO9 along with the isolated TNF related ligands 1 and 3 (TNRL1 and TNRL3) and their active fragments. This patent has been cited much by the Human Genome Sciences, Inc. followed by the Smithkline Beecham Corporation; Genentech, Inc; Biogen Idec Ma Inc; The Uab Research Foundation and Zymogenetics, Inc. which are considered as the active assignee in the field of TNF anticancer targeted drug discovery (Figure 7).

#### Non-patent citation analysis

**Figure 8:**
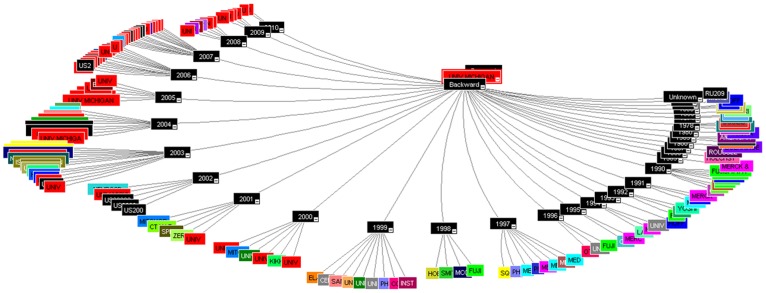
Non-patent citation analysis using Thomson Innovation.

The study would analyse the number of non-patent documents such as review/research articles, letters and other literature in-sights that has been cited in the patent document. The University of Michigan has again reported for the maximum number of non-patent citation with the US patent US8148147B2 (833) and US8497307B2 (231), respectively. Since, the technology of US8148147B2 has already been explained in the cited patents section. The current section would discuss the in-sights of the technology with respect to the US8497307B2 patent (Figure 8). The invention discussed about a family of Aryl guanidine F1F0-ATPase inhibitors and related methods, published in 2013 with 2008 as priority year. The reported aryl guanidine derivatives and their salts, esters, and prodrugs are new and the stereo chemical configuration at a stereocenter in the compound is R and/or S configuration, which act as the mitochondrial F1F0-ATPase inhibitor. The patent was categorised under the composition and inhibitor reporting the literature from the peer reviewed journals like the Tetrahedron, Journal of Biological Chemistry, Journal of Medicinal Chemistry, Nature, PNAS, Bioorganic & Medicinal Chemistry Letters, Medicinal Research Reviews, Journal of Organic Chemistry and Anticancer Research etc. [35].

### Keyword based anticancer technology analysis

**Figure 9:**
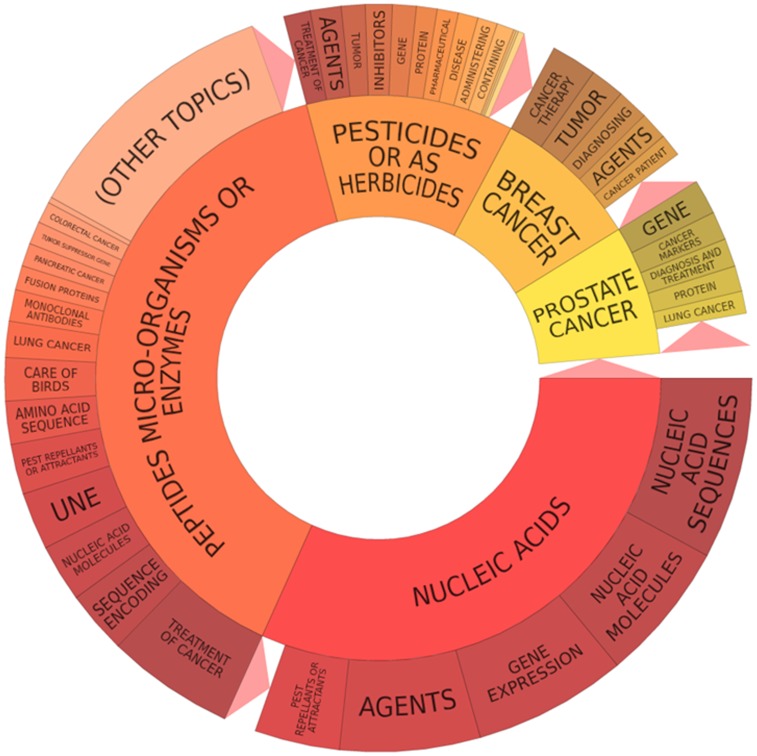
Keyword based technology analysis is shown.

Since, we are dealing with the large patent portfolio of 1584 patent documents; the keyword based analysis was performed for a broad and easy understanding of the various technological areas. The Pat*Base* software was used for this purpose to generate the technology maps and clusters [32]. The technology analysis using keywords is based on the text mining of the patent documents to analyse and report the word, which occurred the maximum number of time. A special artificial intelligence would run behind the software to avoid the common prepositions and retrieve the most significant result. The Figure 9 shows the pie chart representation of the technology cluster of selected portfolio based on keywords present in patent document, representing the peptide microorganism or enzymes as the domination technology next to the nucleic acid and organic/inorganic chemical compounds. The first inner circle represent the first level of categorisation, whereas the outer circle indicates the second level technology category. There are many keyword with respect to the sequence encoding, gene expression, nucleic acid molecules, amino acid sequence, inhibitors, agents, proteins, monoclonal antibodies, diagnosis, treatment and cancer therapy etc.

**Figure 10:**
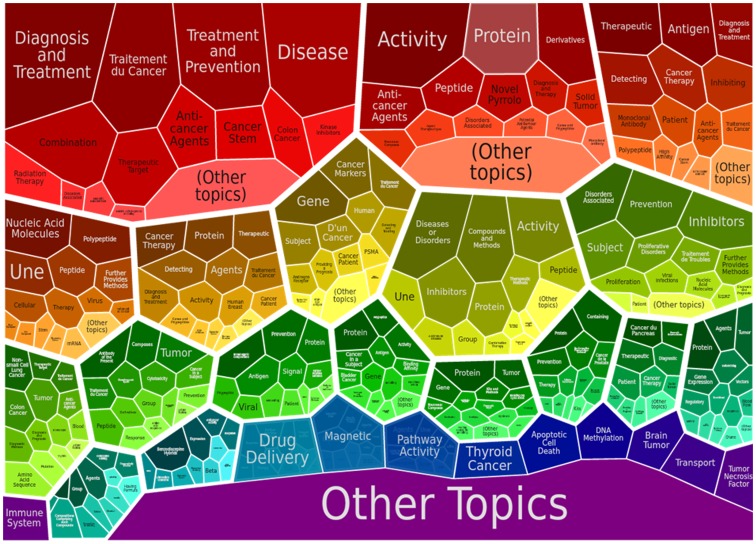
Keyword based cluster map is shown.

The Figure 10, illustrates the second level of the keyword clusters based on the number of technologies in patent portfolio. The first level classification has the following classes which includes treatment of cancer, novel, antibodies, nucleic acid, breast, prostate, pancreatic, thyroid and lung cancer along with the anticancer agents, immune response, small molecules amino acid, cancer cell line, compositions, drug delivery and apoptotic cell death etc. The Figure 10 represents the next level of keyword based clusters which would give a broad idea and help proceed further with the manual categorisation studies. Overall, this keyword based technology categorisation study would give an overview of the various dominating technologies present in the given patent portfolio and narrow down results to specific technology.

### Technology analysis based on manual categorization

**Figure 11:**
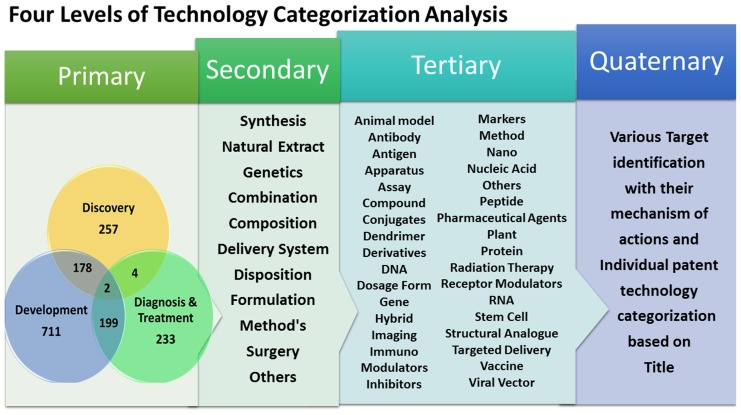
Smart art representation of manual technology categorization.

The earlier keyword based technology analysis would only give a glimpse of the various technologies, but would not categories each and individual patent accordingly. Hence, a manual technology classification was performed on these 1584 patent documents and to categorise them into four different level of classes (Figure 11). The first or primary level of categorisation consist of three main groups of patents such as the discovery group which includes the novel and new findings in the anticancer field followed by development group which includes the additional research on already existing invention such as composition, combination etc. Finally, the diagnosis and treatment group which includes patents related to surgery, methods, apparatus and radiation therapy. The primary level categorisation is illustrated in the form of basic ring interconnected relationship map of smart art, which categorised the patent according to their groups and at the same time segregate the overlapping patents. As per the given details, the maximum number of 711 patents are grouped in the field of development, followed by 257 patents in discovery and 233 in diagnosis and treatment group. The maximum overlapped patents of 199 was reported between development and diagnosis & treatment followed by 178 documents by development and discovery groups. Whereas, the least count of 4 and 2 was reported with respect to discovery and diagnosis and the patents which fall under all the three categories [36]–[40]. The primary level patent categorisation made it clear that anticancer research with respect to development patents are more when compared to discovery and diagnosis.

The second level categorisation would elaborate further the discovery, development and diagnosis group to next class such as the synthetic chemical constituents like inhibitors [41], compounds [42], derivative [43] and hybrids [44] along with the natural extract [45] from plants, micro-organisms [46] and transgenic animal models, new gene sequences [47] are categorised under the discovery group. Whereas, the composition [48], combination for synergic effects [49] along with drug delivery system [50] formulation and disposition are subjected under development group [51]. Finally, the patents which discuss about the surgery [52], radiation therapy [53], apparatus such as imaging and other methods [54] are included under diagnosis and treatment group. Although, the secondary categorisation has segregated the patents into eleven different classes, but still there is a scope of narrow down the study. So, the third level categorisation studies was performed to further differentiate the patents to represent the specific technological area, which can be easily identified and studied. The tertiary manual categorisation has segregated the patents into 34 sub-classes, which uniquely identify each specific technology. These 34 sub-classes include the animal model, antibody, antigen, apparatus, assay [55]–[59], compounds, conjugates, dendrimers, derivatives, DNA [60]–[64], dosage forms, gene, hybrids, imaging technology, immune, modulators [65]–[69], inhibitors, markers, methods, nano particles, nucleic acid [70]–[74], others, peptide, pharmaceutical agents, plants extract, protein [75]–[79], radiation therapy, receptor modulators, RNA, stem cell, structural analogue, targeted delivery, vaccine and viral vectors [80]–[87]. The patents which come under more than one class or sub-class during categorisation studies were specially grouped into multi-class and multi-subclass, respectively. In the same way the patents which cannot categorised in any of the mentioned class or sub-class were grouped under “Others” These 34 sub-classes were finalised after a carful observation and thorough reading of the title, abstract, invention background and claims of each individual patent, which includes a portfolio of 1584 patents; indeed a time consuming and tedious job to perform.

**Figure 12:**
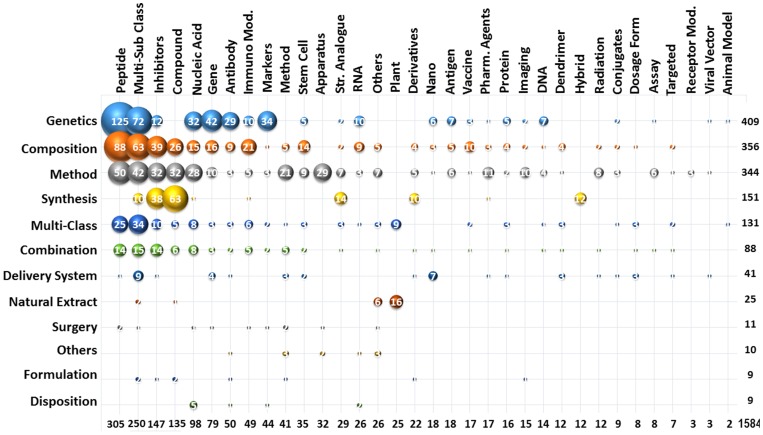
Second and third level technology correlation study represented as bubble graph.

Figure 12 depicts the correlation between various technologies and help identify the domination research fields at the same time discover the white spaces in technology with a scope of future research. The bubble graph was plotted taking secondary class on *X*-axis and tertiary sub-class on *Y*-axis with a bubble density of 50, the greater the bubble size the dominant is the technology. According to the given details, the research in the field of genetic with respect to peptides has dominated all other technologies field reporting 409 and 305, respectively. Whereas, the class composition, methods, multi-class and combination has showed a consistent patent records with most of the tertiary sub-classes accounting 356, 344, 131 and 88 patents, respectively. However, multi-sub class, inhibitors, compounds, nucleic acid, gene, antibody, immune modulators, markers and methods have showed promising patent record of 205, 147, 135, 98, 79, 50, 49, 44 and 41, respectively. Overall, the left half of the bubble chart has covered the major technologies which contribute more than 75% of all patent documents. The right half from sub-class derivatives to animal model and class natural extract to disposition has showed much of the whitespaces in technology correlation map.

Although, each section of the technology cannot be discussed in detail because of the great number of documents. Nonetheless, for an example the insights of two class’s i.e. natural extract and synthesis are explained in detail. There are total 41 patent documents which discuss about natural extracts, but only 25 are visible in the bubble chart and remaining 16 are grouped under other categories. The majority was obtained by the sub-class plant reporting 25 patent documents; 16 under natural extract and 9 under multi-class followed by others, multi-sub class and compound accounting 6, 2 and 1 patents, respectively. The remaining 7 documents are grouped under the 34 documents reported by the multi-class and multi- subclass section accounting to a total of 41 patents. The class plant include the bark of Terminalia arjuna, Himalayan Yew tree Taxus wallichiana, Dichrostachys cinerea, parthenolide its derivatives, Chrysanthemum ethanolic extract, extra-virgin olive oil, European Mistletoe extract, Boswellia species, plant weed Parthenium hysterophorus, Mahanine compound, curcuminoid from Curcuma Longa, leaves of Murraya koeniigii, Piper betel, Tribulus terrestris, extract of Psyllium, Cedrus deodra and angiosperm or gymnosperm, preferably an essential oil plant from the family Lamiaceae such as Mentha piperita, Abies Grandis etc. The other than plant natural extracts include purified extract of snake venom components, extract of lichen Everniastrum cirrhatum, marine Leptolyngbya cyanobacterium, Apratoxin marine natural product and latrunculins a family of natural products and toxins produced by certain sponges, including genus Latrunculia etc. Finally, CSIR patent number US6548086B1 discuss about the pharmaceutical composition comprising extract from plant Cryptolepis buchanani for treating immunodeficiency [88] and the University of Oxford patent WO2007105280A9 explains about the compound epigallocatechin gallate (EGCG) obtained from catechin in tea etc. [89]. All this together should give an overview of the plants and various organism involved in the section natural extracts, which were used to discover and develop the new potential anticancer leads.

Now, considering the secondary class synthesis, which stand at fourth position with 151 patents showing maximum of 63 patent with respect to sub-class compounds followed by the 38 inhibitors, 14 structural analogues, 12 hybrids, 10 derivatives and multi-sub class etc. There are many compound reporting the 1,4-Benzodiazepinone, 1,2,3-triazole containing artemisinin, Diaryl naphthyl methanes, Biarylrhodanine and pyridylrhodanine, C2-fluoro Pyrrolo(2,1-c)(1,4) benzodiazepines, Diarylhydantoin, Trioxane dimer sulfur, Polyketide xanthones, 4-benzoylpiperidine, Substituted 1h-benz(de)isoquinoline-1,3-diones, Dihydrobenzothiepino, dihydrobenzoxepino and tetrahydro benzocyclohepta indoles, 3-hydroxy-2(1h)-pyridinone, Imidazoquinoxalinones, Bismuth dithiocarbamate and Bisphosphonamidate prodrugs etc. The inhibitors includes Thiazolopyrimidines useful as TNF alpha inhibitors, Polypyrrolinone for matrix metalloprotein inhibitors, Polyamines useful as lysine-specific demethylase inhibitors, 5'-substituted adenosynes for S-adenosylmethionine decarboxylase inhibitor, Pyrazole Inhibitors of COX-2 and various kinase and small molecules used for target specific inhibition etc. The structure analogues include Benzo lipoxin, Boronic acid aryl, Pegylated fluorobenzamide, Tubulysin D, 4-amino-2H-pyran-2-one analgous, Illudin analogues, 4beta-1''-((2''-substituted benzoyl) aniline) podophyllotoxin analogues 25-SO2-substituted analogue of 1.alpha.,25-dihydroxyvitamin D3, Substituted 2-(9h-Purin-9-Yl) acetic acid and Boswellic acids, Alpha Galactosylceramide analogues etc. Likewise the hybrids include the Antiproliferative vitamin D3 hybrids and many of the linked pyrrolo(2,1-c) (1,4)benzodiazepine hybrids like C8-linked acridone/acridine, Phenanthrylphenol, Pyrene, Isoxazoline, Diaryl ether and Pyrrolo etc. There are hybrids with respect to Chalcone or Benzothiazole or benzoxazole linked pyrrolo (2,1-c) (1, 4) benzodiazepine hybrids as novel antitumor agents. Finally, the derivatives includes the Combretastatin A-4, Trihydroxy polyunsaturated eicosanoid, Bis-acylated hydroxylamine, Novel Boronic Chalcone, Piperidine, A-substituted phenylpropionic acid, Isthmin, Aziridine aldehydes, Spiro derivatives of Parthenin, aziridine-conjugated amino derivatives, conjugates of artemisinin-related endoperoxides and hydrazone derivatives etc. which are used for the treating cancer. Since, the aim of the article is to identify the position of CSIR India among the top 20 international universities the manual technology analysis was kept limited to only two class of synthesis and natural extract.

### Patent technology analysing using classification codes

The patent technology analysis was performed using international classification systems like Cooperative Patent Classification (CPC), International Patent Classification (IPC) and Drewent’s Chemical Patents Index (CPI). These studies would highlight the various dominating and leading technologies present in the selected field of patent portfolio, which further evaluate the patent categorisation studies which are manually conducted. This study helped in identifying the specific aspects of invention in term of their novelty and innovation using predefined international patent codes which represents a specific area of specialization.

#### Cooperative Patent Classification (CPC)

CPC system is the best classification practice jointly developed and followed by European Patent Office (EPO) and United States Patent and Trademark Office (USPTO). The Table 6 shows the top 20 major CPC classes which are ranked according to their patent count along with their definition. As per the given results, the invention with respect to the medicinal preparations containing peptides (A61K38) have dominated the technology filed followed by the Methods (G01N33) and organic active ingredients (A61K31). Whereas, the next dominating technology identified was the processes involving enzymes, nucleic acids or micro-organisms (C12Q1) followed by medicinal preparations containing antigens or antibodies (A61K2039, A61K39) and Immunoglobulins [84], e.g. monoclonal or polyclonal antibodies (C07K16). There are many patents in the field of genetic engineered hybrid peptides (C12N15) and pharmacogenomics and oligonucleotides (C12Q2600) and gene therapy (A61K48). Overall the CPC technology classification analysis has made it clear that the technology area with respect to proteins, peptides and immunology topic like antibody and antigen are reported as dominating filed of research areas.

**Table 6:** Cooperative Patent Classification (CPC) analysis.

CPC	Count	CPC Code Definition
A61K38	381	Medicinal preparations containing peptides
G01N33	371	Investigating or analysing materials by specific Methods
C07K14	366	Peptides having more than 20 amino acids; Gastrins; Derivatives thereof
A61K31	350	Medicinal preparations containing organic active ingredients
C12Q1	295	Measuring or testing processes involving enzymes, nucleic acids
C07K16	221	Immunoglobulins, e.g. monoclonal or polyclonal antibodies
C12Q2600	209	Oligonucleotides characterized by their use, Pharmacogenomics, i.e. genetic variability in individual responses to drugs and drug metabolism
C12N15	172	Screening of peptide libraries presented on the surface of microorganisms/Genetic engineering processes for obtaining hybrid peptides
A61K2039	169	Medicinal preparations containing antigens or antibodies
A61K39	160	Medicinal preparations containing antigens or antibodies
A61K47	122	Medicinal preparations characterised by the non-active ingredients used
A61K48	116	Medicinal preparations containing genetic material which is inserted into cells of the living body to treat genetic diseases; Gene therapy
G01N2333	110	Assays involving biological materials from specific organisms
A61K45	108	Immunological preparations stimulating the reticulo-endothelial system
C07K2317	101	Antibody isolated from natural sources
C07K2319	101	Fusion peptides and immunoglobulin+a non-antibody protein
C12N9	96	Peptides with enzymatic activity
G01N2500	87	Screening for compounds of potential therapeutic value
C12N2310	84	Structure or type of the nucleic acid
G01N2800	74	Detection or diagnosis of diseases

#### International Patent Classification (IPC)

IPC system classify the patents and utility models into various technology areas which they pertain. It is one of the oldest patent classification system established in year 1971 by the Strasbourg Agreement, which amend regularly by the experts in IPC Committee. Table 7 illustrates the top 20 IPC technology classification based on the IPC class codes and ranked according to their patent count. The antineoplastic agent specific towards leukaemia and metastasis (A61P35) has occupied the first position in the technological ranking. However, A61P35 being the broad category filed which can be either placed in chemistry or biology, the further classification help identified medicinal preparations containing organic active ingredients and peptides as the dominating technology area resembling the CPC classification results. Furthermore, patents with respect to methods (G01N33) and testing processes involving enzymes or micro-organisms and compositions (C12Q1) from the development group have occupied the next dominating technology level. The next dominating technology is genetically engineered DNA, RNA and vectors and their preparation thereof (C12N15) followed by the medicinal preparations containing antigens or antibodies (A61K39). Finally, the technologies like the cell line studies or cell therapy (C12N5), gene therapy (A61K48), immune modulation (C07K16) and enzymes composition and thereof (C12N9) are reported as the leading technologies of the public funded anticancer patents.

**Table 7:** International Patent Classification (IPC) analysis.

IPC	Count	IPC Code Definitions
A61P35	846	Antineoplastic agents specific for leukaemia and metastasis
A61K31	764	Medicinal preparations containing organic active ingredients
A61K38	593	Medicinal preparations containing peptides
G01N33	569	Investigating or analysing materials by specific Methods
C12Q1	534	Measuring or testing processes involving enzymes or micro-organisms; Compositions therefor; Processes of preparing such compositions
C12N15	524	Mutation or genetic engineering; DNA or RNA concerning genetic engineering, vectors, e.g. plasmids, or their isolation, preparation or purification;
A61K39	452	Medicinal preparations containing antigens or antibodies
C07K14	421	Peptides having more than 20 amino acids; Gastrins; Derivatives thereof
C12N5	357	Undifferentiated human, animal or plant cells, e.g. cell lines; Tissues; Cultivation or maintenance thereof; Culture media therefor
A61K48	350	Medicinal preparations containing genetic material which is inserted into cells of the living body to treat genetic diseases; Gene therapy
C07K16	305	Immunoglobulins, e.g. monoclonal or polyclonal antibodies
C07H21	274	Compounds containing two or more mononucleotide units having separate phosphate or polyphosphate groups e.g. nucleic acids
A61P43	242	Drugs for specific purposes, not provided for in groups A61P 1/00-A61P 41/00
A61K45	241	Medicinal preparations containing active ingredients not provided for in groups A61K 31/00-A61K 41/00
C12P21	185	Preparation of peptides or proteins (single-cell protein C12N 1/00)
A61P37	173	Drugs for immunological or allergic disorders
A61K35	164	Medicinal preparations containing material or reaction products thereof with undetermined constitution
A61P31	154	General protective or antinoxious agents
C12N9	150	Enzymes, e.g. ligases; Proenzymes; Compositions thereof; Processes for preparing, activating, inhibiting, separating, or purifying enzymes
A61K47	131	Medicinal preparations characterised by the non-active ingredients used, e.g. carriers, inert additives

#### Chemical Patents Index (CPI) Manual Codes

CPI system is a Derwent's manual coding system, where trained analyst categorises the various technologies into specific field of distinguished inventions. Table 8 depicts the data of top 20 CPI code along with their definitions, the data suggest that the cancer related drugs (B14-H01) as the dominating technology. The next leading technology was reported by the recombinant protein/polypeptide production (D05-H17) and diagnosis of tumours, cancer (D05-H17) followed by the medical preparation involving organic active ingredients (B14-L06) such as inhibitors, antagonist, antimetabolite etc. The fourth position is occupied by the antibody and antigens (B04-G, D05-H11) followed by viral vectors including plasmid, cosmids, transposons viral vectors (B04-E08, D05-H12E). The CPI class D05-H12 and B12-K04F have reported preparation containing DNA, cDNA, transfer vectors, RNA and tests involving DNA, hybridisation probes etc. The remaining field of technologies include the cells, microorganisms, hosts, cell lines, tissue culture (B04-F01), primers, probes (B04-E05) and protein/polypeptide of undefined origin (B04-N02).

**Table 8:** Chemical Patents Index (CPI) manual codes analysis.

DWPI	Count	DWPI Class Definition
B14-H01	1029	Cancer Related Drugs
D05-H17	730	Recombinant protein/polypeptide production
B12-K04A1	489	Diagnosis of tumours, cancer
B14-L06	331	Antagonist/inhibitor/antimetabolite general and other
B04-G	309	Antibody defined in terms of antigen
B04-E08	301	Vectors, plasmids, cosmids, transposons Viral vectors
D05-H12E	270	Vectors Includes viral vectors (e.g. Baculovirus vectors, phagemids), plasmid vectors, cosmids and transposons.
D05-H11	254	Antibodies
D05-H12	252	DNA, cDNA, transfer vectors, RNA
B12-K04F	240	Tests involving DNA, hybridisation probes etc.
B11-C08E	222	Biological procedures for testing general
B14-C03	207	Antiinflammatory general
B11-C07A	203	Antigen - antibody reaction general
B04-F01	189	Cells, microorganisms, transformants, hosts, cell lines, tissue general
D05-H14	180	Recombinant cells Host cells (prokaryotic and eukaryotic)
B04-N02	176	Animal protein/polypeptide (No sequence)
B04-N04	169	Protein/polypeptide of undefined origin (No sequence)
B12-K04E1	169	Drug discovery process
B04-E05	162	Primers, probes
B14-H01B	161	Antiproliferative, inhibitor of cell division, cytostatic

The overall patent technology analyses using various international classification codes such as CPC, IPC and CPI have suggested that in all the three technology classification the area with respect to genetics such as peptides, gene therapy, nucleic acids, micro-organisms, viral vectors and immunology topic like antibody and antigen are dominating field of area next to the medical preparation containing organic active ingredients such as inhibitors, derivative, compounds and hybrids etc. As the results from technology analysis are in supporting with the manual four level of technology categorisation study, this study should further authenticate the generated patent landscape report.

### Anticancer target analysis

**Figure 13:**
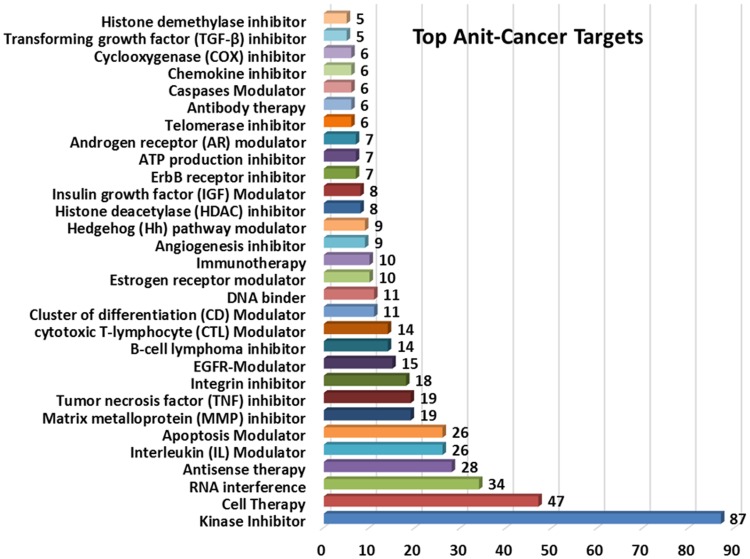
Top anticancer targets of public funded research organization.

In an ever changing cancer research the innovative ways to explore the biological mechanism of cancer through targets identification has played a key role toward the drug discovery. Over a period of time there are many new molecules designed, synthesised, extracted and formulated to bind specifically to therapeutic targets so as to regulate the cell proliferation and survival. In an effort to identify and screen the various mechanisms which act as potential anticancer targets, we also performed a target analysis on the selected patent portfolio. Although, there are few patents which specify the enzyme, receptor and pathway way inhibitors, but many of the patents account the anticancer activity through generalised cell growth inhibitors or assay methods. As the technology based categorisation has already proved peptides as dominating research area there are many patents which show the mechanism of action as gene therapy, vaccine and cell growth inhibitors which were omitted to collect only the list of specific receptors. The Figure 13 depicts the top 30 anticancer targets ranked according to their patent count, represented in the form of bar graph plotting targets on *X*-axis and patent count on *Y*-axis.

The kinase inhibitor stood first as potential anticancer target reporting more than 87 patents. There are many type of kinases inhibitor available which includes Abelson oncogene (Abl) tyrosine kinase; Aurora A kinase; Ctrl/Dictyostelium tyrosine kinase 1 (DPYK1); Axl tyrosine kinase; B-Raf kinase; Breakpoint cluster region gene and cellular Abl (BCR-ABL) kinase; Bruton's tyrosine kinase (Btk); Cyclin dependent kinase (CDK); Deoxycytidine kinase (dCK); Dictyostelium kinase-1 (DICTY-I); GFR family tyrosine kinase; Endothelial tyrosine kinase (Etk); Extracellular receptor kinase (ERK); FMS-like tyrosine kinase 3 (FLT3); Glycogen synthase kinase 3 (GSK3); HUNK protein kinase; Intracellular urokinase plasminogen activator (uPA); Leucine-rich repeat kinase 2 (Lrrk-2); lkB-alpha kinase; Maternal-embryonic-leucine-zipper-kinase; Mer tyrosine kinase (Mertk); Mitogen-activated protein kinase 14 (MAPK14); Modulator of c-Jun N-terminal kinase (JNK); MOK kinase; P21 activated kinases (PAK)/serine/threonine-protein kinase(STE20; SRPK1; Sgk494); PDZ-binding (PB) kinase; Phosphoinositide 3-kinase (PI3K); Protein kinase C (PKC); Proto-oncogene serine/threonine-protein (Pim)-1 kinase; Pyruvate dehydrogenase kinase; Raf-kinase inhibitor protein (RKIP); RhoA kinase (ROCK); Rous sarcoma protein tyrosine kinase (Src); Sphingosine Kinase; T-LAK cell-originated protein (TOP) kinase; Type II hexokinase activity inhibitor; Urokinase type plasminogen activator receptor (uPAR) and Yak/Yrk (tyrosine-phosphorylation regulated kinase) inhibitors etc. [90]–[94].

The cancer cell therapy, a process of using the cellular material such as tissue from embryos or foetuses of animals is injected into patient to treat cancer. The cell therapy stand second followed by drugs acting as RNA interface and antisense therapy, a synthesized nucleic acid used to treat a variety of diseases including cancer. The other anticancer receptor modulators included Interleukin (IL), Cytotoxic T-lymphocyte (CTL), Epidermal growth factor receptor (EGFR), Cluster of differentiation (CD), Insulin growth factor (IGF), Androgen receptor (AR), Estrogen receptor(ER) and Hedgehog (Hh) pathway modulator. Now coming to the various inhibitors which are reported as potential anticancer targets include Matrix metalloprotein (MMP), Tumor necrosis factor (TNF), Integrin inhibitor, B-cell lymphoma, Histone deacetylase (HDAC), Transforming growth factor (TGF-β) and Histone demethylase (H3-K specific) inhibitors. There are many other mechanisms which contributed as potential anticancer targets such as apoptosis inducers, immunotherapy, DNA binder, angiogenesis inhibitor, Antimetastatic inducer, caspases modulator and antibody therapy. The other inhibitors include the ErbB receptor, ATP production, Telomerase, Chemokine, Cyclooxygenase (COX) inhibitor, inhibitor etc.

Apart from the mentioned list of various targets, there are many more mechanism and targets which are reported for anticancer activity, such as Toll like receptor (TLR) agonist, Methyltransferase modulator, G-Protein-Antagonist, Cannabinoid receptor modulator, Epithelial membrane protein-2 (EMP 2) Modulator and various enzyme and receptor inhibitors like Cysteine protease, Angiotensin, Lysine demethylase, Lipoxygenase, Fatty acid synthase (FAS) inhibitor, Aromatase, Cytokine, Topoisomerase, Ephrin receptors (Ephs), Cathepsin, Cytochrome CYP24 and Cyclophilin inhibitors etc. Cancer being the most deadly disease with a constant exploration and new finding of various targets added up every year can make the list exhaustive. The current target analysis has reported almost 50 targets that have been mentioned and used by the top international universities and public funded organisations as potential anticancer targets.

## Future Insights

The raw data of 1584 patents was collected and a proper meaning was deduced through a four level manual categorisation studies to convert the data into information. The primary, secondary and tertiary technology categorisation have provided with the necessary information to identify the various technologies. Further, the information was analysed through a correlation study performed on secondary class and tertiary sub-class to drive the knowledge in the form of bubble chart which gives further in-sights of anticancer patents. The various anticancer research technology area were examined and reason out peptide as the dominating technology followed by the anticancer treatment using gene therapy, immunotherapy, chemotherapy, antisense therapy and cell therapy etc. Likewise, one can deduce the immense information through the technology correlation bubble chart, not only to identify the dominating technologies but also to screen the new research areas by cross checking one technology field with other so as to come to a logical conclusion. For an instant, if we consider the secondary class “compositions”, there is a scope of research in the field of composition vs hybrids and composition vs plant extracts. Similarly, the targeted therapy has showed patents with respect to combination, methods, compositions and delivery system but not formulations, so the targeted therapy vs formulation is another promising area to work. The bubble chart areas showing only few patents such as one or two are considered as upcoming technologies and vacant areas as whitespaces, which can be explored further to identify the key concepts of that research area. There is a huge score of research to be performed with respect to discovering new apparatus for treating cancer, targeted therapy or drug delivery systems and surgery methods etc. Overall, the technology correlation bubble chart would provide with an information which can be used for future cancer research. In addition, the international patent technology classification study using IPC, CPC and CPI codes has further validated the manual technology categorisation studies. Although, there are many targets reported by the selected patent portfolio, only the top 50 were listed out indicating various kinases inhibitors as active target.

The current article has also examined the role of Indian CSIR among top 20 international universities and the research finding have ranked CSIR at seventh position reporting University of California as the leading assignee. Whereas, the inventor analysis has listed out top 29 anticancer research scientist and Dr. Nakamura, Yusuke from University of Tokyo has taken the lead, currently working as oncology professor of medicine in the University of Chicago. Two of the CSIR scientist Dr. Kamal, Ahmed and Dr. Saxena, Ajit, Kumar has made it to the top 29 inventors list. The collaboration network analysis has reported University of California as leading university to show maximum internal as well as external collaborations. The geographical analysis revealed United States of America as the leading country to file maximum patents followed by Australia and Canada. The patent trend analysis has reported the current trend of anticancer patent filing and granting patterns, along with individual year wise patent distribution based on priority, application and publication years. The citation map analysis has identified stem cell research, polypeptide therapy and organic preparation as the core technology area through forward, backward and non-patent citation maps. In addition, an in-depth technology assessment was performed using keyword based analysis and four level technology categorisation studies and explained in detail discussing about various anticancer targets as well. To conclude, although the Indian CSIR has not been ranked at top international level, but the research finding in the area of anticancer field has reported CSIR as one of the potential global competitor based on the patent landscape report generated.
